# A new contribution to the raptorial ciliate genus *Lacrymaria* (Protista: Ciliophora): a brief review and comprehensive descriptions of two new species from Changjiang Estuary

**DOI:** 10.3389/fmicb.2023.1259653

**Published:** 2023-11-06

**Authors:** Jin Tang, Gongaote Zhang, Junqi Guo, Lingxuan Luo, Jiamei Jiang, Hongbo Pan

**Affiliations:** ^1^Shanghai Universities Key Laboratory of Marine Animal Taxonomy and Evolution, Shanghai Ocean University, Shanghai, China; ^2^Institute of Evolution and Marine Biodiversity, Ocean University of China, Qingdao, China; ^3^National Demonstration Center for Experimental Fisheries Science Education, Shanghai Ocean University, Shanghai, China

**Keywords:** Lacrymariidae, new combination, new species, Changjiang Estuary, SSU rRNA gene, taxonomy

## Abstract

Ciliates serve as excellent indicators for water quality monitoring. However, their utilization is hindered by various taxonomic confusions. The ciliate genus *Lacrymaria* Bory de Saint-Vincent, 1824 is commonly found in different aquatic habitats, but its taxonomy has been sparsely investigated using state-of-the-art methods. This study investigated two new *Lacrymaria* species from Nanhui Wetland, Shanghai, China, using living observation, protargol staining, and molecular phylogeny methods. *Lacrymaria songi* sp. nov. is 180–340 × 20–25 μm in size and possesses 12–16 somatic kineties, 1 terminal contractile vacuole, 2 macronuclear nodules, and 2 types of rod-shaped extrusomes. *Lacrymaria dragescoi* sp. nov. is distinguished from its congeners by its cell size of 210–400 × 25–35 μm, 14–17 somatic kineties, 1 terminal contractile vacuole, 1 macronucleus, and 2 types of rod-shaped extrusomes. Phylogenetic analyses based on SSU rRNA gene sequences indicate that Lacrymariidae is monophyletic but *Lacrymaria* is not. Additionally, a brief review of the genus *Lacrymaria* is provided in this study. We suggest that *L. bulbosa* Alekperov, 1984, *L. lanceolata* Kahl, 1930, and *L. ovata* Burkovsky, 1970 be removed from the genus and propose *Phialina lanceolata* nov. comb. and *Phialina ovata* nov. comb. for the latter two.

ZooBank registration: Present work: urn:lsid:zoobank.org:pub:CDFB1EBD-80BD-4533-B391-CEE89F62EDC4 *Lacrymaria songi* sp. nov.: urn:lsid:zoobank.org:act:417E7C2D-DAEC-4711-90BB-64AB3CD2F7D5 *Lacrymaria dragescoi* sp. nov.: urn:lsid:zoobank.org:act:8778D6B0-1F2E-473C-BE19-3F685391A40D.

## 1. Introduction

Ciliates are excellent indicators for water quality monitoring and play a vital role in the aquatic microbial food web (Wang et al., 2022; Weisse and Montagnes, 2022). *Lacrymaria* ciliates are common raptorial microorganisms found in aquatic habitats worldwide (Kahl, 1930; Dragesco, 1960, 1965; Rajter et al., 2019; Wang et al., 2019). They can be easily identified by the bubble-like head located at the front end of their body, which is covered by short oblique kineties (Lynn, 2008). The family Lacrymariidae de Fromentel 1876 includes four genera, namely, *Lacrymaria* Bory de Saint-Vincent, 1824, *Pelagolacrymaria* Foissner, 1999, *Phialina* Bory de Saint-Vincent, 1824, and *Phialinides* Foissner, 1988 (Lynn, 2008). In contrast to other well-studied haptorians, such as pleurostomatids and spathidiids, research on the Lacrymariidae is limited, and its phylogeny remains unresolved (Foissner, 1988; Foissner and Xu, 2007; Rajter et al., 2019; Pan et al., 2020; Wu et al., 2021, 2022; Chi et al., 2022; Zhang G. et al., 2022a,b,c).

*Lacrymaria* Bory de Saint-Vincent, 1824 is the largest and oldest genus of the family Lacrymariidae. It is distinguished from its relatives by the presence of a retractable neck (Foissner, 1983). However, for a long time, its closest related genus *Phialina* was considered as its synonym, which results in the affiliation of most *Lacrymaria* species needing to be re-considered. Several *Lacrymaria* species have already been transferred to *Phialina*, *Lagynus*, or *Pelagolacrymaria* in recent studies (Supplementary Table S1; Foissner, 1983, 1987; Song and Wilbert, 1989; Sola et al., 1990; Foissner et al., 1995, 1999, 2002; Wang et al., 2019; Jiang et al., 2023). Since the descriptions of most *Lacrymaria* species are rough and superficial, the species delimitation is understudied. Recent phylogenetic analysis of Haptoria based on either single gene locus or multiple gene loci has shown that *Lacrymaria* is not monophyletic (Wu et al., 2017; Huang et al., 2018; Rajter et al., 2019; Wang et al., 2019). However, this conclusion is not confident because only 3 out of 53 nominal *Lacrymaria* species have molecular information, and the DNA sequence that detached from *Lacrymaria* in gene trees is not reported along with morphometrics (Huang et al., 2018; Rajter et al., 2019).

Recent studies on haptorian ciliates in China have revealed a high diversity of the order Pleurostomatida (Liu et al., 2017; Pan et al., 2020; Wu et al., 2021, 2022; Zhang G. et al., 2022a,b,c, 2023). However, little attention has been given to the family Lacrymariidae. A project on ciliate fauna conducted in Changjiang Estuary has led to the discovery of various new or rarely known ciliates (Chen et al., 2022; Han et al., 2022; He et al., 2022; Zhang Z. et al., 2022a,b). As a new contribution, we investigated the phylogeny and taxonomy of two new *Lacrymaria* species, namely, *L. songi* sp. nov. and *L. dragescoi* sp. nov., using integrative methods including live observation, silver staining, and DNA sequencing. Additionally, we provide a brief review of the genus *Lacrymaria*.

## 2. Materials and methods

### 2.1. Sample collection and cultivation

**Figure 1:**
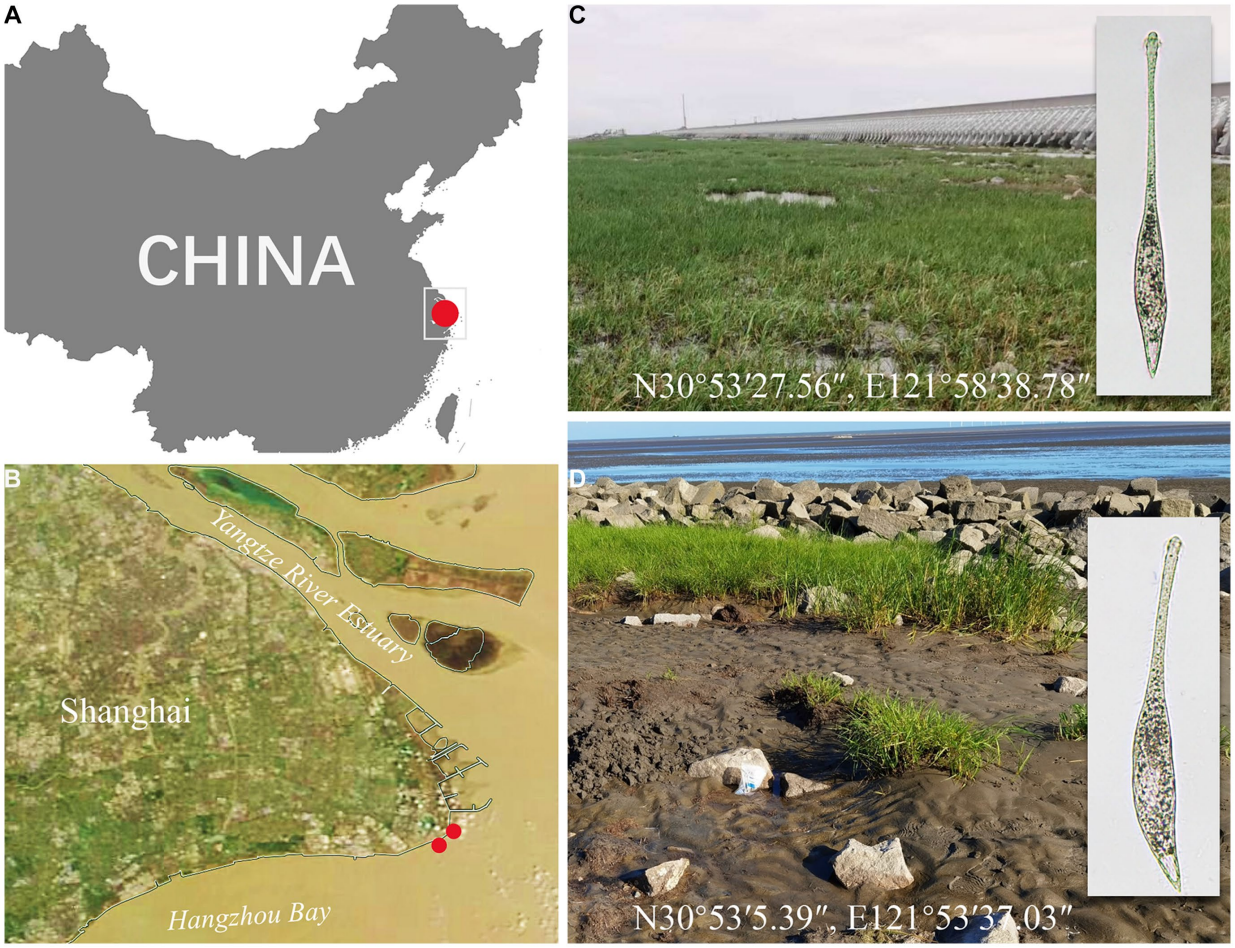
Sampling sites **(A–D)**. **(A)** A part of the map of China showing the location of Shanghai. **(B)** A satellite image of Shanghai showing the location of Nanhui Wetland. **(C)** Photograph of the sampling site in a tidal flat of Nanhui Wetland (N30°53′27.56″, E121°58′38.78″) from where *Lacrymaria songi* sp. nov. was collected. **(D)** Photograph of the sampling site in the tidal flat of Nanhui Wetland (N30°53′5.39″, E121°53′37.03″) from where *Lacrymaria dragescoi* sp. nov. was collected.

*Lacrymaria songi* sp. nov. and *Lacrymaria dragescoi* sp. nov. were both collected on 28 September 2022 from two adjacent sites of Nanhui Wetland (N30°53′27.56″, E121°58′38.78″), Shanghai, China (Figure 1). For the habitat of *L. songi* sp. nov., the water temperature was 23.6°C, the pH was 7.43, the concentration of dissolved oxygen (DO) was 4.22 mg/L, and the salinity measured in the Petri dish was 17‰; for the habitat of *L. dragescoi* sp. nov., the water temperature was 23.6°C, the pH was 7.61, the DO was 5.47 mg/L, and the salinity measured in the Petri dish was 20‰. All environmental parameters, except salinity, were measured *in situ*, and the salinity was measured when *Lacrymaria* species were detected in the raw cultures.

After the samples were transported to the laboratory, raw cultures were immediately established in Petri dishes with rice grains to enrich the growth of bacteria, serving as food for the ciliates. *L. songi* sp. nov. and *L. dragescoi* sp. nov. were detected after 3 weeks.

### 2.2. Morphological observation

Live cells were observed using bright field and differential interference contrast microscopy (Olympus BX53) at magnifications of 100–1,000. The ciliary pattern was revealed using the protargol preparation method (Wilbert, 1975). The protargol reagent was manually synthesized following the method described by Pan et al. (2013). Counts and measurements of stained specimens were performed at a magnification of 1,000, and drawings were made at the same magnification with the aid of a camera lucida. Terminology and systematics are explained by Lynn (2008) and Vd’ačný et al. (2011).

### 2.3. DNA extraction, PCR amplification, and DNA sequencing

Five cells of each species were isolated from the raw cultures using sterile micropipettes and washed at least five times with filtered (0.22 μm) habitat water to remove contaminants. Genomic DNA was extracted by a DNeasy Blood & Tissue Kit (Qiagen, Hilden, Germany) using one-quarter of the volume recommended by the manufacturer’s instructions as described by Shao et al. (2023). The SSU rRNA gene was amplified by PCR using the primers 18S-F (5’-AAC CTG GTT GAT CCT GCC AGT-3′) and 5.8S-R (5′- TAC TGA TAT GCT TAA GTT CAG CGG-3′) (Medlin et al., 1988; Sogin, 1989). The cycling parameters were as follows: an initial denaturation of 3 min at 95°C, followed by 30 cycles of 30 s at 95°C, 20 s at 56°C, and 1.5 min at 72°C, with a final extension of 5 min at 72°C. The PCR products were, then, purified, cloned, and sequenced, following the method described by Chen et al. (2022). The sequencing data were assembled using SeqMan v7.1 (DNAStar), and sequence similarities were calculated using BioEdit v.7.2.5 (Hall, 1999).

### 2.4. Phylogenetic analyses

The SSU rRNA gene sequences of *Lacrymaria songi* sp. nov. and *L. dragescoi* sp. nov. were aligned with 52 other sequences downloaded from GenBank, including three metopids as outgroup taxa, namely, *Clevelandella panesthiae* (KC139719), *Metopus palaeformis* (AY007450), and *Nyctotherus ovalis* (AJ222678). The alignment was performed using the MUSCLE algorithm on the Web Server Guidance^1^ with default settings (Sela et al., 2015). Maximum likelihood (ML) analyses were conducted using RAxML-HPC2 (Stamatakis, 2014) on XSEDE v.8.2.11 on the CIPRES Science Gateway^2^ under the GTRGAMMA model with 1,000 bootstraps. Bayesian inference (BI) analysis was performed using MrBayes v.3.2.7 (Ronquist et al., 2012) on the same platform under the GTR + I + Γmodel, which was selected by jModelTest 2 via the Akaike Information Criterion (Darriba et al., 2012). Markov chain Monte Carlo simulations were run for 1,000,000 generations, and trees were sampled every 100 generations with a burn-in of 2,500 trees (25%). The tree topology was visualized using Figtree v1.4.4 (Rambaut, 2018).

The support of the dataset for competing phylogenetic hypotheses was evaluated using the approximately unbiased (AU) test to test the monophyly of the genus *Lacrymaria* (Shimodaira and Hasegawa, 2001). The site-wise likelihoods for the resulting constrained topology and the non-constrained ML topology were calculated using RAxML v.8.2.11 under a partitioned GTR + GAMMA model (Yang, 1996; Stamatakis, 2014). The same model was used to estimate the site likelihoods for those trees prior to conducting the AU test. The scores of each constraint tree were compared with the unconstrained ML result using the AU test option implemented in CONSEL (Shimodaira and Hasegawa, 2001).

## 3. Results

Subclass Haptoria Corliss, 1974.

Family Lacrymariidae de Fromentel, 1876.

Genus *Lacrymaria* Bory de Saint-Vincent, 1824.

### 3.1. *Lacrymaria songi* sp. nov.

#### 3.1.1. Diagnosis

Size: approximately 180–340 × 20–25 μm *in vivo*. Body shape: highly variable depending on the state of contraction, ranging from a vase-shaped body in the contracted state to fusiform to clavate in the extended state. Neck: flexible, occupying half of the body length and up to two-thirds of body length when swimming, and neck beating 92 times/min when preying. Extrusomes have two types: type I—approximately 10 μm long, rod-shaped, mostly arranged in bundles, scattered in main body, and attached to oral bulge; type II—approximately 4 μm long, rod-shaped, scattered in main body and 12–16 somatic kineties. Single terminally located contractile vacuole. Two macronuclear nodules. Brackish habitat.

#### 3.1.2. Type locality

A muddy tidal flat of Nanhui Wetland (N30°53′27.56″, E121°58′38.78″), Shanghai, China.

#### 3.1.3. Type specimens

A protargol slide (registration no. TJ2022090805-1) with the holotype circled in black ink and one paratype slide (TJ2022090805-2) are deposited in the Laboratory of Protozoology, Ocean University of China.

#### 3.1.4. Dedication

The species is named in honor of Prof. Weibo Song, Ocean University of China, in recognition of his outstanding contribution to Ciliatology.

#### 3.1.5. SSU rRNA gene sequence

The SSU rRNA gene sequence of *L. songi* sp. nov. has been deposited in GenBank (accession no. OR689566) with 1,641 bp long and GC content of 42.41%.

#### 3.1.6. Description

**Figure 2:**
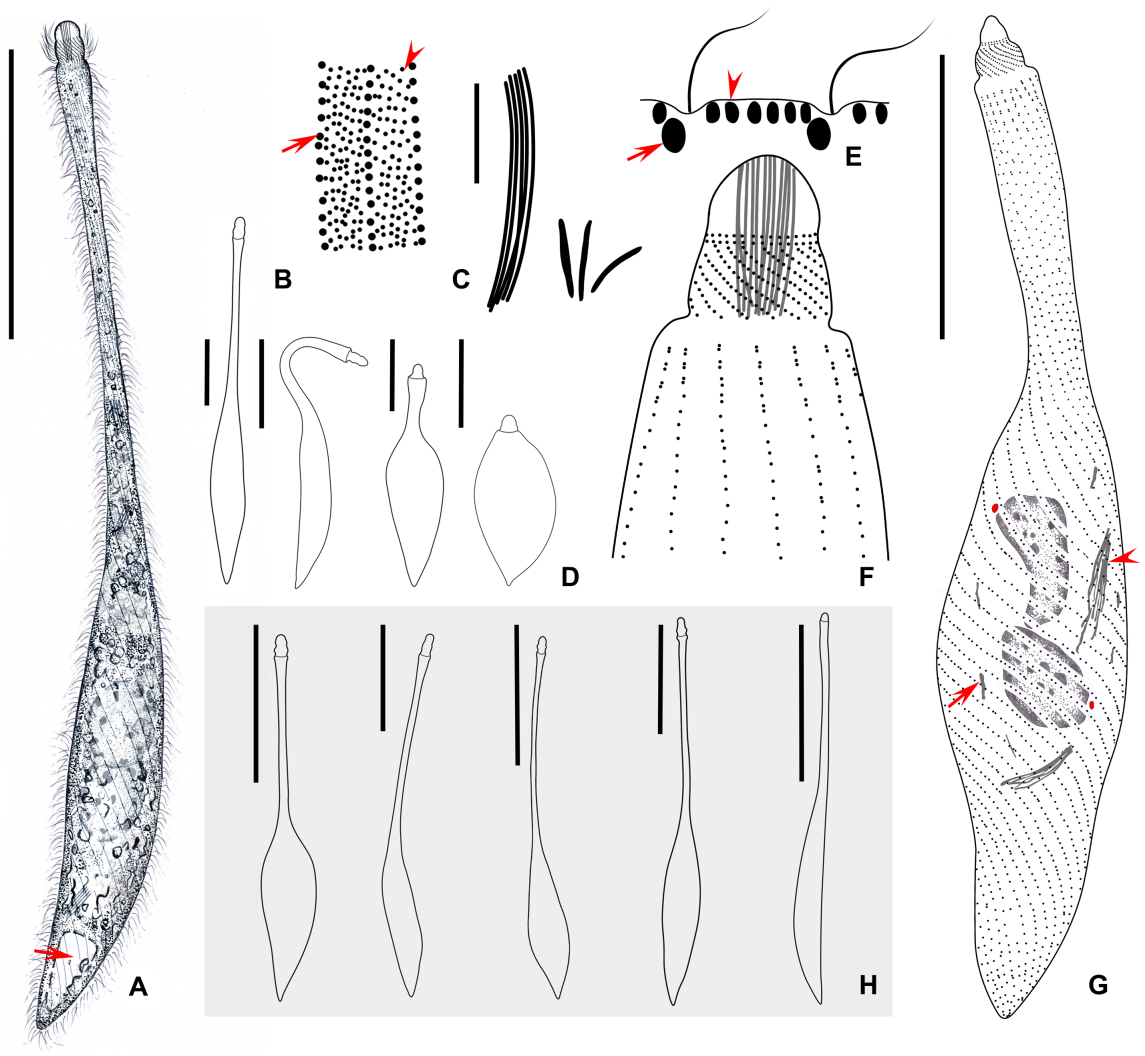
**(A–H)** Morphology of *Lacrymaria songi* sp. nov. from life **(A,B,D,E,H)** and after protargol staining **(C,F,H)**. **(A)** Typical extended individual arrow points to the contractile vacuole. **(B)** Two types of cortical granules; type I is marked by an arrowhead and type II is marked by an arrow. **(C)** Two types of extrusomes. **(D)** Shape variants in one individual. **(E)** Arrangement of cortical granules on the cell surface; type I is marked by an arrowhead and type II is marked by an arrow. **(F)** The anterior end of the ciliary pattern. **(G)** Ciliary pattern of the holotype specimen; arrowhead points to a long type of extrusome, arrow points to a short type of extrusome, and red dots indicate micronuclei. **(H)** Shape variants in different individuals. Scale bars: 100 μm in **(A,H)**; 5 μm in **(C)**; 50 μm in **(D,G)**.

**Figure 3:**
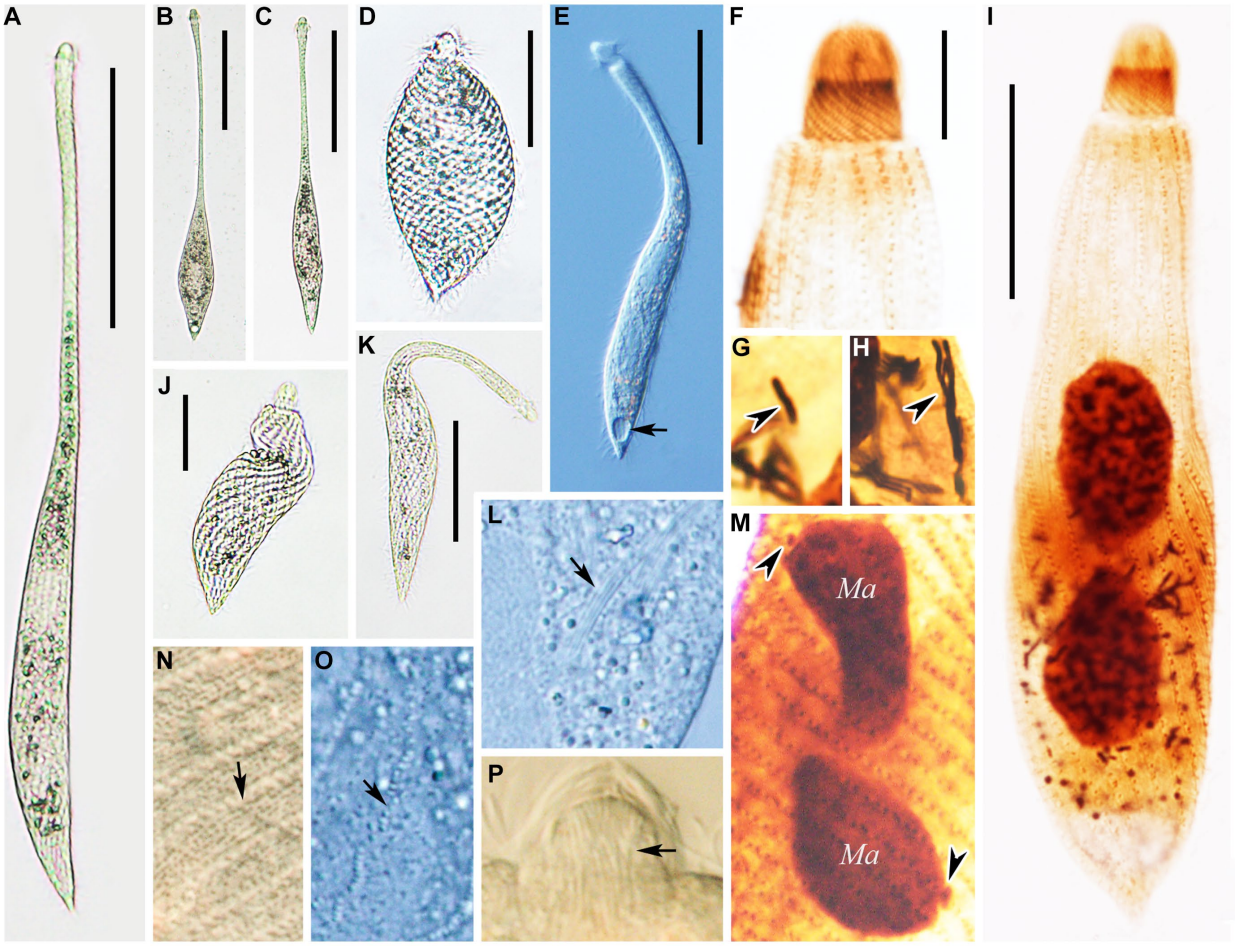
Photomicrographs of *Lacrymaria songi* sp. nov. from life **(A–E,I–K,M–O)** and after protargol staining **(F–H,L)**. **(A)** A representative individual. **(B,C)** Different free-swimming individuals show shape variants. **(D)** A completely contracted individual. **(E,J,K)** Individuals in different contraction states; arrow in **(E)** points to the contractile vacuole. **(F)** Details of the anterior portion showing the anterior somatic kineties. **(G)** Details of the cytoplasm; arrowhead points to a short extrusome. **(H)** Details of the cytoplasm; arrowhead points to long extrusomes. **(I)** A representative specimen showing ciliature and nuclear apparatus. **(L)** Details of the cytoplasm; arrow points to extrusomes. **(M)** Details of the cytoplasm; arrowheads point to micronuclei. **(N,O)** Details of cytoplasm in the middle of the body; arrows point to two different cortical granules. **(P)** Head structure arrows point to extrusomes. Ma, macronuclear nodules. Scale bars: 100 μm in **(A–C)**; 30 μm in **(D)**; 50 μm in **(E,H,J)**; 23 μm in **(I)**; 12 μm in **(F)**.

Cell: highly contractile, when fully extended cell size *in vivo* approximately 180–340 × 20–25 μm and length:width ratio of 11:1 (Figures 2A, 3A–C) and when contracted, cell size approximately 65–102 × 28–40 μm and length:width ratio of 3:1 (Figures 2D, 3D). Body shape fusiform to clavate with flexible neck, occupying half of body length, and up to two-thirds of body length when swimming. The posterior end tapered and tail-like when free swimming but vase-shaped with neck retracting into trunk and the posterior end broadly tapered when contracted (Figures 2A,D,H, 3A,E,J,K).

Two ovoidal macronuclear nodules centrally located with a filament connected to each other, each approximately 12–20 × 8–13 μm *in vivo* and approximately 10–32 × 6–21 μm after protargol staining (Figures 2G, 3I,M and Table 1). Two micronuclei detected only in 1 out of 30 stained individuals, respectively, located at subapical of each macronuclear nodule (Figure 2G, 3M). However, micronucleus not detected in live cells. Single contractile vacuole terminally located, variable in shape, ranging from rounded to obovate, approximately 11 × 17 μm during diastole, pulsating every 5 min (Figure 3E). Two types of extrusomes: type I approximately 10 μm long, rod-shaped, straight or slightly curved, mostly arranged in bundles, scattered in main body, and attached to oral bulge; type II approximately 4 μm long, rod-shaped, straight or slightly curved, scattered in main body (Figures 2C,F, 3G,H,L,P). Both types of extrusomes easily detected after protargol staining but only type I detectable *in vivo*. Two types of colorless cortical granules: type I dot-like, approximately 0.4 μm *in vivo*, densely arranged in five or six rows between kineties in peripheral region of cortex, this character may vary slightly with body contraction; type II dot-like to oval-shaped, approximately 0.8 μm *in vivo*, only distributed along somatic kineties deep in cortex (Figures 2B,E, 3N,O). Cytoplasm colorless or grayish, containing numerous globular granules (<4 μm in diameter) in trunk, rendering neck hyaline and trunk opaque (Figure 3L). Locomotion usually by swimming fast with neck swinging; when preying, neck extends forward and backward and retracts rapidly, beating approximately 92 times per minute, whereas trunk moves in a small range (Supplementary Video S1).

**Table 1:** Morphometric characteristics of *Lacrymaria songi* sp. nov. (the upper line) and *L. dragescoi* sp. nov. (the lower line) based on protargol stained specimens.^a^

Characteristics	Min	Max	Mean	*M*	SD	CV	*n*
Body length	88.0	187.0	129.9	123.0	28.2	21.7	30
	104.0	194.0	143.0	142.0	21.4	14.9	28
Body width	15.0	39.0	25.8	26.0	6.0	23.3	30
	28.0	58.0	37.3	35.0	7.5	20.0	28
Body length: width, ratio	3.1	7.7	5.2	5.0	1.0	19.5	30
	2.1	5.4	4.0	4.0	0.8	19.3	28
Head height	9.0	14.0	11.4	11.0	1.2	10.6	30
	7.0	11.0	9.3	9.0	1.0	11.1	28
Head width	5.0	10.0	7.4	7.0	1.4	19.4	30
	6.0	10.0	7.5	7.5	1.2	15.3	28
Anterior body end to Ma, distance	31.0	115.0	52.3	47.5	17.7	33.9	30
	42.0	105.0	65.2	64.5	14.0	21.4	28
Ma, length^b^	10.0	32.0	17.7	15.5	6.4	36.5	30
	20.0	42.0	30.4	29.0	6.6	21.5	28
Ma, width^b^	6.0	15.0	9.9	8.5	3.0	30.0	30
	7.0	24.0	14.2	14.0	4.3	30.3	28
Extrusome length, type I	7.0	16.0	11.2	11.0	2.0	17.8	21
	9.0	20.0	13.2	13.0	2.6	19.9	26
Somatic kineties, number	12.0	16.0	13.4	13.0	1.0	7.4	30
	14.0	17.0	15.1	15.0	0.7	4.6	28
Dikinetids in anterior portion of somatic kinety, number	3.7	5.2	4.4	4.3	0.4	9.1	28
	4.3	5.8	5.1	5.1	0.4	7.9	27
Somatic ciliary rows, distance in between	0.3	1.0	0.8	0.8	0.2	23.0	30
	2.8	5.5	4.2	4.0	0.7	17.4	28
Kinetids, distance in between	1.8	4.2	2.7	2.7	0.6	21.2	30
	0.5	0.9	0.7	0.7	0.1	15.8	28
Kinetids per somatic kinety, number	80.0	184.0	125.7	121.0	22.0	17.5	28
	118.0	223.0	156.6	156.0	22.4	14.3	26

All measurements in μm. ^a^CV, coefficient of variation (%); M, median; Ma, macronuclear nodule; Max, maximum; Mean, arithmetic mean; Min, minimum; n, number of individuals investigated; SD, standard deviation. ^b^Data for *Lacrymaria songi* sp. nov. are from the anterior macronuclear nodule.

Somatic cilia approximately 9 μm long, densely arranged in 12–16 (13 on average) somatic kineties. Somatic kineties slightly spiral *in vivo* when cell extended but broadly spiral in contracted individuals and protargol preparations (Figures 3A,D,I). Each kinety composed of 3–6 (4.4 on average) dorsal brush dikinetids anteriorly (Figures 2F,G, 3F), and 80–184 somatic monokinetids posteriorly with some dikinetids irregularly interspersed (Figure 2F and Table 1). Head kineties densely spirally arranged, with cilia approximately 10 μm long. Circumoral kinety is composed of approximately 28 circumoral dikinetids (Figures 2E,F).

### 3.2. *Lacrymaria dragescoi* sp. nov.

Syn. *Lacrymaria olor sensu* Dragesco, 1966, pr. p. Figure 11b.

#### 3.2.1. Diagnosis

Size: approximately 210–400 × 25–35 μm *in vivo*. Body shape: highly variable depending on the state of contraction, ranging from a vase-shaped body in the contracted state to fusiform to clavate in the extended state. Neck: flexible, occupying half of the body length, and accounting for two-thirds of the body length when swimming, and neck beating 30 times/min when preying. Extrusomes have two types; type I—approximately 13 μm long, rod-shaped, mostly arranged in bundles, scattered in main body and attached to oral bulge; type II—approximately 3 μm long, rod-shaped, scattered in main body, and 14–17 somatic kineties. Single terminally located contractile vacuole. One macronuclear nodule. Brackish habitat.

#### 3.2.2. Type locality

A tidal flat of Nanhui Wetland (N30°53′5.39″, E121°53′37.03″), Shanghai, China.

#### 3.2.3. Type specimens

A protargol slide (registration no. TJ2022090807-1) with the holotype circled in black ink and one paratype slide (TJ2022090807-2) are deposited in the Laboratory of Protozoology, Ocean University of China, Qingdao, Shandong, China.

#### 3.2.4. Dedication

The species is named in honor of Prof. Jean Dragesco, in recognition of his contributions to Ciliatology.

#### 3.2.5. SSU rRNA gene sequence

The SSU rRNA gene sequence of *L. dragescoi* sp. nov. has been deposited in GenBank (accession no. OR689567) with 1,642 bp long and GC content of 42.75%.

#### 3.2.6. Description

**Figure 4:**
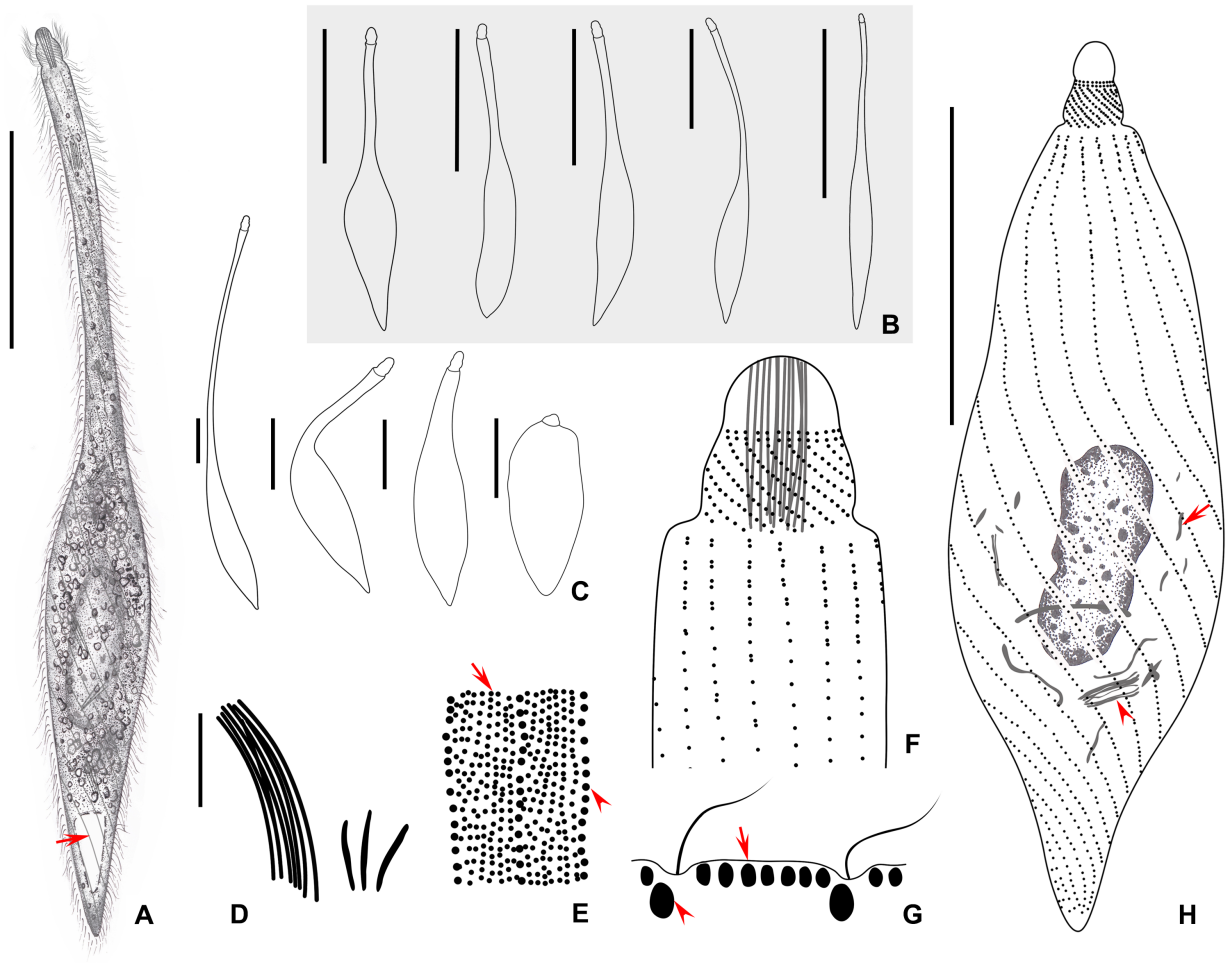
**(A–H)** Morphology of *Lacrymaria dragescoi* sp. nov. from life **(A–C,E,G)** and after protargol staining **(D,F,H)**. **(A)** A typical extended individual; arrow points to the contractile vacuole. **(B)** Shape variants between different individuals. **(C)** Shape variants of the same individual. **(D)** Two types of extrusomes. **(E)** Two types of cortical granules; type I is indicated by red arrow and type II is indicated by red arrowheads. **(F)** Anterior end of ciliary pattern showing the head kineties and somatic kineties. **(G)** Arrangements of the cortical granules on the cell surface; type I is indicated by red arrow and type II is indicated by red arrowheads. **(H)** Ciliary pattern of the holotype specimen; arrowhead points to the long type of extrusomes and arrow points to the short type of extrusomes. Scale bars: 100 μm in **(A,B)**; 50 μm in **(C)**; 5 μm in **(D)**; 25 μm in **(H)**.

**Figure 5:**
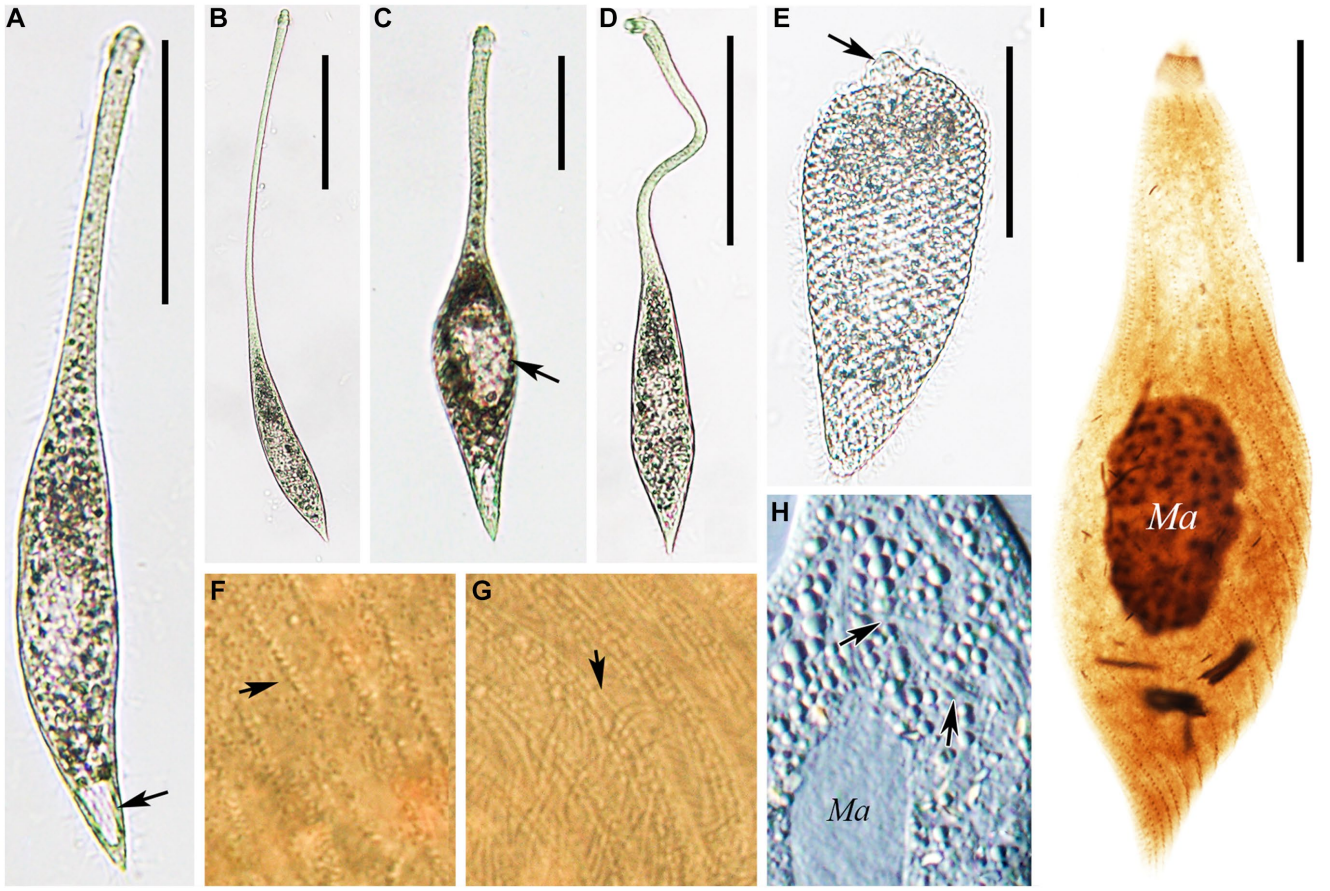
Photomicrographs of *Lacrymaria dragescoi* sp. nov. from life **(A–H)** and after protargol staining **(I)**. **(A)** A representative individual; arrow points to the contractile vacuole. **(B)** An extended individual. **(C)** An individual that is seeking prey; arrow points to a large food vacuole. **(D)** An individual with a neck somewhat extended for searching for food. **(E)** A completely contracted individual, arrowhead infers to the head. **(F,G)** Details of the cell surface in the middle of the body; arrows point to the two different cortical granules. **(H)** Details of the cytoplasm; arrows point to extrusomes. (I) The holotype specimen showing the ciliature and nuclear apparatus. Ma, macronucleus. Scale bars: 100 μm in **(A–D)**; 50 μm in **(E)**; 30 μm in **(I)**.

Cells: highly contractile; when fully extended, cell size of approximately 210–400× 25–35 μm *in vivo* and length:width ratio of 10:1 (Figures 4A, 5A,B); when contracted, cell size of approximately 100–170 × 36–44 μm and length:width ratio of 3:1 (Figures 4C, 5E). Body shape fusiform to clavate with flexible neck, occupying half of the body length and accounting for two-thirds of the body length when swimming. The posterior end tapered and tail-like when free swimming but vase-shaped with neck retracting into trunk, and posterior end sharply rounded when contracted (Figures 4A–C, 5A,D,E).

Nuclear apparatus centrally located, comprising one oval-shaped macronucleus, approximately 17–25 × 6–9 μm *in vivo* and approximately 20–40 × 7–24 μm after protargol staining (Figures 4H, 5H,I and Table 1). Micronucleus undetected *in vivo* or after protargol staining. Single contractile vacuole terminally located, variable in shape, ranging from rounded to obovate, approximately 17 × 12 μm during diastole, and pulsating every 3 min (Figures 4A, 5A). Two types of extrusomes: type I approximately 13 μm long, rod-shaped, straight or slightly curved, mostly arranged in bundles, scattered in main body, and attached to oral bulge; type II approximately 3 μm in size, rod-shaped, straight or slightly curved, scattered in main body (Figures 4D,F,H, 5H,I). Both types of extrusomes easily detected after protargol staining but only type I detectable *in vivo*. Two types of cortical granules: type I dot-like, approximately 0.4 μm *in vivo*, in the peripheral region of cortex densely arranged in seven or eight rows between kineties, this character may vary slightly with body contraction; type II dot-like to oval-shaped, approximately 0.7 μm *in vivo*, only distributed along somatic kineties deep in cortex; both types of cortical granules colorless (Figures 4E,G, 5F,G). Cytoplasm colorless or grayish, containing numerous globular granules (< 2.6 μm in diameter) in trunk, rendering neck hyaline and trunk opaque (Figure 5H). Locomotion usually by swimming fast with neck swinging; when preying, neck extends forward and backward and retracts rapidly, beating approximately 30 times per minute, whereas trunk moves in a small range (Supplementary Video S2).

Somatic cilia approximately 8 μm long, densely arranged in 14–17 (15 on average) somatic kineties. Somatic kineties slightly spiral *in vivo* when cell extended but broadly spiral in contracted individuals and protargol preparations (Figures 4H, 5A,E,I). Each kinety composed of 3–6 (5.1 on average) dorsal brush dikinetids anteriorly and 118–223 somatic monokinetids posteriorly with some dikinetids irregularly interspersed (Figures 4F,H, 5I and Table 1). Head kineties densely spirally arranged, with cilia approximately 8 μm long. Circumoral kinety composed of approximately 30 circumoral dikinetids (Figures 4F,I).

### 3.3. Sequence comparison and molecular phylogeny

**Figure 6:**
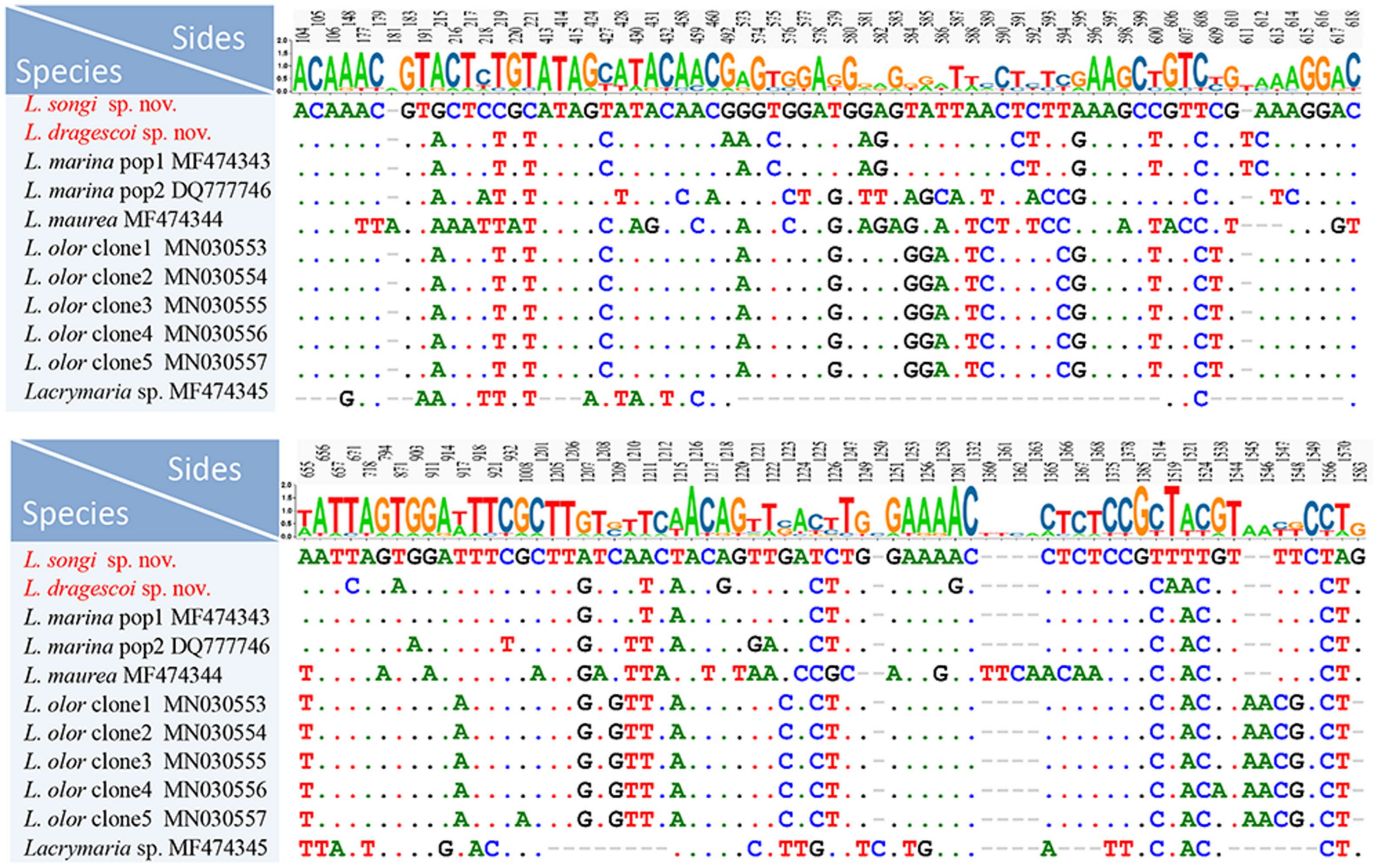
Nucleotide differences between *Lacrymaria songi* sp. nov., *Lacrymaria dragescoi* sp. nov., and other *Lacrymaria* species. Two new sequences in our present study are the first two rows. Numbers indicate the position of nucleotides. Missing sites are indicated by dashes (−).

The nucleotide similarities of the SSU rRNA gene sequences between *Lacrymaria* species range from 94.45 to 99.62%. *L. songi* sp. nov. differs from congeners except for *L*. *dragescoi* sp. nov. by 25–66 nucleotides, with sequence identities ranging from 95.69 to 98.43%. *L. dragescoi* sp. nov. differs from congeners except for *L. songi* sp. nov. by 6–60 nucleotides, with sequence identities ranging from 96.08 to 99.62% (Figure 6).

**Figure 7:**
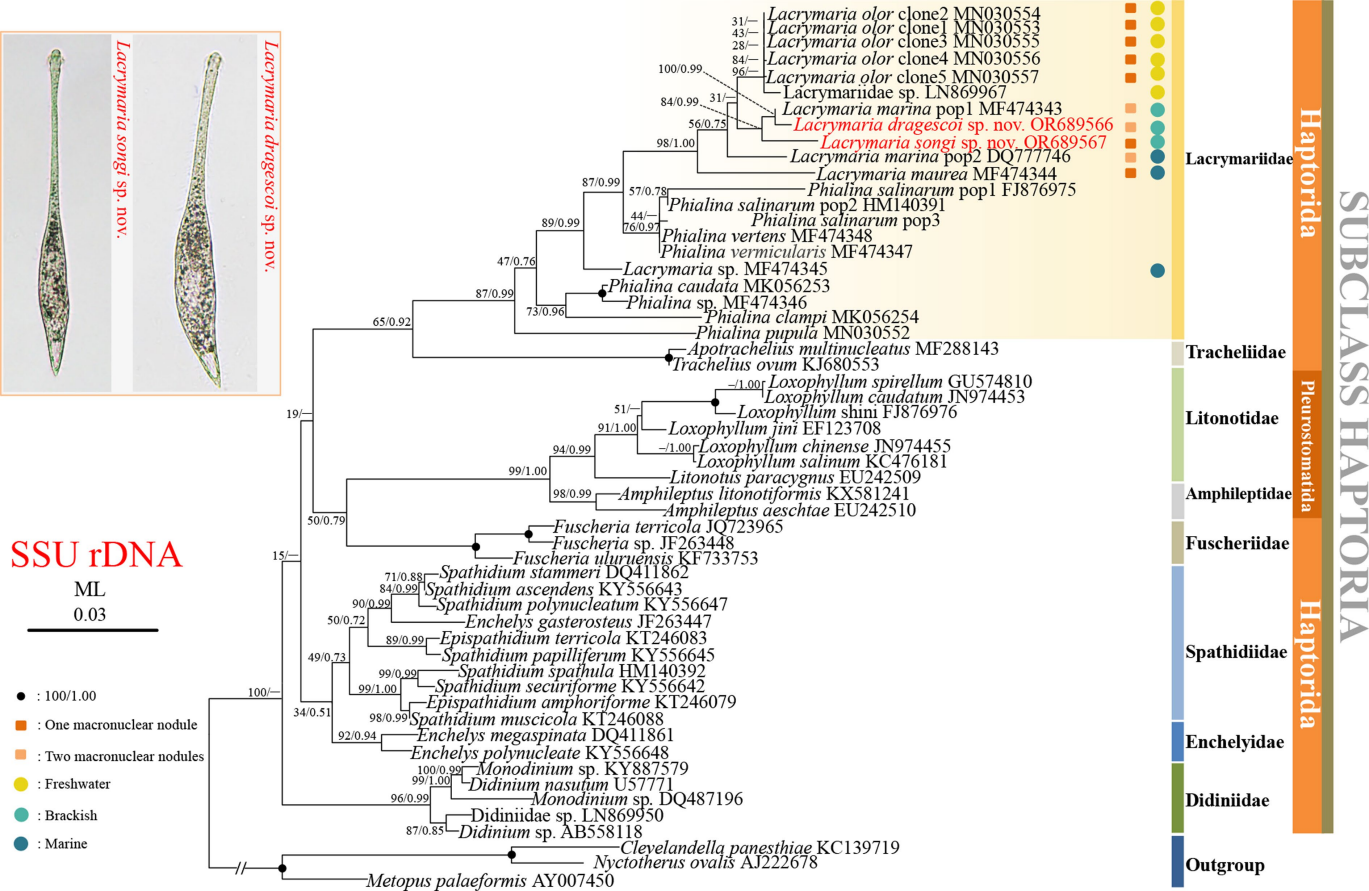
Phylogenetic tree based on SSU rRNA gene sequences, displaying the phylogenetic positions of *Lacrymaria songi* sp. nov. and *L. dragescoi* sp. nov. (red). Numbers near branches denote bootstrap values for maximum likelihood (ML) and posterior probabilities for Bayesian inference (BI). “–” indicates the disagreement between ML and BI trees. GenBank accession numbers are provided after species names. The scale bar corresponds to three substitutions per 100 nucleotide positions.

The topologies of the ML and BI trees were basically congruent with varying levels of support; therefore, only the ML tree is presented in Figure 7. As shown in the ML tree, *Lacrymaria songi* sp. nov. and *L. dragescoi* sp. nov. fall in the core of *Lacrymaria*, and the family Lacrymariidae is recovered as a monophyletic group (Figure 7). The genus *Lacrymaria* is non-monophyletic with *Lacrymaria* sp. (MF474345) groups with *Phialina*. The AU test also refutes the monophyly of *Lacrymaria* (AU > 0.05). In the ML tree, *L. dragescoi* sp. nov. groups with *Lacrymaria marina* pop1 (MF474343) with high support (ML/BI, 100%/0.99), forming a sister clade to *L. songi* sp. nov. Then, they depict a monophyletic group that is sister to a clade formed with very weak support by *Lacrymaria olor* clone 1–5 (MN30553–MN30557) and Lacrymariidae (LN869967).

## 4. Discussion

### 4.1. A brief review of the genus *Lacrymaria* Bory de Saint-Vincent, 1824

*Lacrymaria* is easily recognized by its long, contractile, and flexible neck. However, the history of *Lacrymaria* is marked by confusion. Both *Lacrymaria* and its relative *Phialina* were originally defined based on misinterpreted oral features. *Phialina* has also experienced abandonment and re-activation (Bory de Saint-Vincent, 1824; Foissner, 1983). Currently, there are approximately 53 nominal species within the genus *Lacrymaria* (Supplementary Table S1). However, only 12 species have been investigated through live observation and silver staining. Furthermore, those *Lacrymaria* species without infraciliature data have mostly not been rediscovered since their original reports raised questions about their validation and affiliations.

*Lacrymaria bulbosa* Alekperov, 1984, *L. lanceolata* Kahl, 1930, and *L. ovata* Burkovsky, 1970 do not possess a contractile neck (Kahl, 1930; Gelei, 1954; Burkovsky, 1970a; Alekperov, 1984). This indicates that they should be removed from *Lacrymaria*. There are three genera of *Lacrymaria* with an acontractile neck, namely, *Pelagolacrymaria* Foissner et al., 1999, *Phialina* Bory de St. Vincent, 1824, and *Phialinides* Foissner, 1988. In terms of ciliary patterns, *L. lanceolata* and *L. ovata* lack a monokinetid circle and a dikinetid circle between the head and the trunk. Therefore, they should be assigned to the genus *Phialina* as new combinations, i.e., *Phialina lanceolata* nov. comb. and *Phialina ovata* nov. comb. However, the affiliation of *L. bulbosa* cannot be determined presently due to its unknown ciliary pattern.

*Lacrymaria sapropelica* Kahl, 1927 and *L. urnula* Kahl, 1930 both have a furrow encircling the neck-like region, which indicates that they should belong to the family Lagynusidae (Jiang et al., 2023). Since their ciliary patterns have not been investigated, further investigation is needed to determine their exact affiliations, particularly through protargol staining and SSU rRNA gene sequencing.

The molecular phylogeny of *Lacrymaria* was initially investigated by sequencing the SSU rRNA gene of *L. marina* Kahl, 1933 (Gao et al., 2008). Subsequently, Rossi et al. (2016), Huang et al. (2018), and Rajter et al. (2019) sequenced nine new SSU rRNA gene sequences of the genus and found that *Lacrymaria* was likely a non-monophyletic genus. However, none of those sequences were reported with the morphological data, which cast doubt on these results.

### 4.2. Comments on *Lacrymaria songi* sp. nov.

Previous studies indicate that the following characteristics can be used for the circumscription of *Lacrymaria* species: the number of somatic kineties, the number of macronuclear nodules, the number and position of micronuclei, the number and position of contractile vacuoles, and characteristics of the extrusomes (Foissner, 1983; Dragesco and Dragesco-Kernéis, 1986; Song and Wilbert, 1989; Foissner et al., 1995; Rajter et al., 2019; Wang et al., 2019).

*Lacrymaria olor* (Müller, 1786) Bory de Saint-Vincent, 1824 resembles *L. songi* sp. nov. in body size, the number of somatic kineties, and the shape of the posterior end (Foissner et al., 1995). However, *L. olor* can be clearly distinguished from *L. songi* sp. nov by the location of the micronucleus (a micronucleus located between the two macronuclear nodules vs. two micronuclei located at the subapical of each macronuclear nodule) and habitat (freshwater vs. brackish water).

In terms of body length and shape, four species should be compared with *Lacrymaria songi* sp. nov., namely, *L. clavarioides* Alekperov, 1984, *L. inflata* Vuxanovici, 1959, *L. maurea* Dragesco, 1965, and *L. metabolica* Bünger, 1908 (Table 2). Among them, only *L. inflata* has a terminally located contractile vacuole, which is similar to *L. songi* sp. nov., but the shape of the posterior end (round vs. pointed) and the habitat of *L. inflata* (freshwater vs. brackish water) are different from those of *L. songi* sp. nov. Compared with *L. songi* sp. nov., *L. clavarioides* has more somatic kineties (20–25 vs. 12–16) and lives in a freshwater habitat (vs. brackish water), *L. maurea* has a rather short neck (vs. occupying up to two-thirds of the body length when swimming), and *L. metabolica* has a round-shaped tail (vs. pointed) and lives in freshwater (vs. brackish water) (Kahl, 1930; Vuxanovici, 1959; Dragesco, 1965; Alekperov, 1984).

**Table 2:** Comparison of *Lacrymaria songi* sp. nov. with congeners that possess two macronuclear nodules.^a^

Species	Body length, μm	No. of SK	CV, position	Contractible neck	Shape of posterior end	Habitat	Data source
*L. songi* sp. nov.	178–338	12–16	Terminal	Present	Pointed	Brackish water	Present work
*L. australis*	46–60	6	Subterminal	Present	Pointed	Freshwater	Foissner and O'donoghue (1990)
*L. binucleata*	30–50	8–12	Subterminal	Present	Pointed	Freshwater	Song and Wilbert (1989)
*L. clavarioides*	250–300	20–25	Subterminal	–	Pointed	Freshwater	Alekperov (1984)
*L. inflata*	–	12–18	Terminal	Present	Round	Freshwater	Vuxanovici (1959)
*L. issykkulica*	40–60	12–15	Terminal	–	Pointed	Freshwater	Alekperov and Asadullayeva (1997)
*L. maurea*	280	–	Subterminal	–	Pointed	Marine	Dragesco (1965)
*L. metabolica*	55–100	–	Subterminal	–	Round	Freshwater	Kahl (1930)
*L. olor*	300–500	13–16	Subterminal and middle	Present	Pointed	Freshwater	Foissner et al. (1995)
*L. parva*	35–40	8–10	–	–	Round	Freshwater	Vuxanovici (1962)
*L. pulchra*	50–80	4–5	Terminal	Present	Pointed	Freshwater	Wenzel (1953)

^a^SK, somatic kineties; CV, contractile vacuoles. –, data not available.

There are five more species possessing two macronuclear nodules, namely, *Lacrymaria australis* Foissner, 1990, *L. binucleata* Song and Wilbert, 1989, *L. issykkulica* Alekperov, 1997, *L. parva* Vuxanovici, 1962, and *L. pulchra* Wenzel, 1953. They can be separated from *L. songi* sp. nov. by the body size, the number of somatic kineties, and the position of contractile vacuoles (for details, refer to Table 2; Kahl, 1930; Wenzel, 1953; Vuxanovici, 1962; Song and Wilbert, 1989; Foissner and O'donoghue, 1990; Alekperov and Asadullayeva, 1997).

### 4.3. Comments on *Lacrymaria dragescoi* sp. nov.

In terms of body length and shape, a single contractile vacuole, and a single macronucleus, 14 species should be compared with *Lacrymaria dragescoi* sp. nov. These species are *L. acuminata* Vuxanovici, 1962, *L. acuta* Kahl, 1933, *L. affinis* Bock, 1952, *L. delamarci* Dragesco, 1960, *L. elongata* Vuxanovici, 1963, *L. filiformis* (Maskell, 1886) Foissner, 1983, *L. foliacea* Vuxanovici, 1962, *L. lagynus* Gelei, 1954, *L. marina* Kahl, 1933, *L. rotundata* Dragesco, 1960, *L. salinarum* Kahl, 1928, *L. trichocystus* Dragesco, 1960, *L. versatilis* (Quennerstedt, 1865) Borror, 1963, and *L. vitrea* Vuxanovici, 1959.

*Lacrymaria dragescoi* sp. nov. closely resembles *L. marina* Kahl, 1933 regarding the general morphology, such as body size, body shape, habitat, and characteristics of extrusomes. However, *L. dragescoi* sp. nov. can be clearly distinguished from *L. marina* by the number of somatic kineties (14–17 vs. 19–23; on average 15 vs. 20) (Table 3 and Song and Packroff, 1997).

**Table 3:** Comparison of *Lacrymaria dragescoi* sp. nov. with congeners that possesses single macronucleus.^a^

Species	Body length, μm	No. of SK	CV, position	Contractile neck	Shape of posterior end	Habitat	Data source
*L. dragescoi* sp. nov.	211–398	14–17	Terminal	Present	Pointed	Brackish water	Present work
*L. acuminata*	125	–	Posterior quarter	–	Pointed	Freshwater	Vuxanovici (1962)
*L. acuta*	180–200	36–40	Subterminal	Present	Pointed	Brackish water	Kahl (1933)
*L. affinis*	230–250	–	Subterminal	–	Pointed	Marine	Bock (1952)
*L. cohni*	70–90	12	Subterminal	Present	Round	Marine	Buitkamp and Wilbert (1974)
*L. conifera*	50–70	18–20	–	–	Round	Marine	Burkovsky (1970b)
*L. delamarci*	140–180	–	Terminal	–	Round	Marine	Dragesco (1960)
*L. elongata*	–	–	Anterior and terminal	–	Round	Freshwater	Vuxanovici (1963)
*L. exigua*	40–70	–	Anterior half and terminal of the body	Present	Round	Freshwater	Vuxanovici (1962)
*L. filiformis*	120–160	10	Subterminal	Present	Pointed	Freshwater	Foissner (1983)
*L. flagellifera*	60	17	Terminal	–	Round	–	Gellért (1957)
*L. foliacea*	–	–	Subterminal	–	Pointed	Freshwater	Vuxanovici (1962)
*L. fusus*	60	–	Posterior third	–	Pointed	Freshwater	Vuxanovici (1962)
*L. kahli*	600–1,000	–	Terminal	Present	Pointed	Marine	Dragesco (1960)
*L. lagynus*	100–150	28–30	Terminal	Present	Round	Freshwater	Gelei (1954)
*L. lata*	32	7–8 on one side	Terminal	–	Round	Freshwater	Vuxanovici (1962)
*L. marina*	200–300	19–23	Terminal	Present	Pointed	Marine	Song and Packroff (1997)
*L. minima*	60	–	Subterminal	–	Pointed	–	Kahl (1930)
*L. minuta*	45	22–24	Subterminal	–	Pointed	Marine	Dragesco (1963)
*L. nana*	40–60	13	Subterminal	Present	Pointed	Freshwater	Song and Wilbert (1989)
*L. oblonga*	70	6–8 on one side	One in posterior quarter, one in anterior third	–	Round	Freshwater	Vuxanovici (1962)
*L. perlucida*	45	–	–	–	Round	Freshwater	Vuxanovici (1963)
*L. pumilio*	40–80	10	Terminal	–	Round	Freshwater	Foissner (1983)
*L. rotundata*	80–150	30	Terminal	–	Round	Marine	Dragesco (1960)
*L. salinarum*	–	–	Subterminal	–	Pointed	Marine	Kahl (1930)
*L. subsphaerica*	30–50	8–9 on one side	Terminal	–	Round	Freshwater	Vuxanovici (1962)
*L. trichocystus*	500	38	Subterminal	–	Pointed	Marine	Dragesco (1960)
*L. vaginifera*	30–40	7–9	Terminal	Present	Round	Freshwater	Song and Wilbert (1989)
*L. versatilis*	200–250	20	Terminal	–	Pointed	Marine	Borror (1963)
*L. vitrea*	–	–	Terminal	–	Round	Freshwater	Vuxanovici (1959)

^a^CV, contractile vacuoles; SK, somatic kineties. –, data not available.

*Lacrymaria delamarci*, *L. lagynus*, *L. rotundata*, *L. vitrea,* and *L. versatilis* have a single terminally located contractile vacuole, which is the same as *L. dragescoi* sp. nov. However, the tail shape of the former four species is round (vs. pointed in *L. dragescoi* sp. nov.), and *L. lagynus* and *L. rotundata* are clearly distinguished from *L. dragescoi* sp. nov. by the number of somatic kineties (28–30, 30 vs. 14–17). Moreover, *L. vitrea* differs from *L. dragescoi* sp. nov. by the habitat (freshwater vs. brackish water) (Gelei, 1954; Vuxanovici, 1959; Dragesco, 1960; Borror, 1963). Unlike *L. dragescoi* sp. nov., *L. versatilis* has a wider neck when extended (two-thirds of body width vs. less than one-third of body width in *L. dragescoi* sp. nov.), which clearly distinguishes them (Borror, 1963).

Other similar congeners with two macronuclear nodules can be distinguished from *Lacrymaria dragescoi* sp. nov. by the location or the number of contractile vacuoles, the habitat, and the number of somatic kineties (for details, refer to Table 3).

*Lacrymaria olor sensu* Dragesco, 1966, pr. p. (Figure 11b) resembles *L. dragescoi* sp. nov. in habitat and most morphological characteristics (Dragesco, 1966). However, the new species is smaller (211–398 μm long vs. 300–500 μm long) and has fewer somatic kineties (14–17 vs. 16–20). Since these differences cannot clearly separate them, we tentatively assign *L. olor sensu* Dragesco, 1966, pr. p. (Figure 11b) as a synonym of *L. dragescoi* sp. nov. (Dragesco, 1966).

### 4.4. Phylogenetic analyses

With the addition of *Lacrymaria songi* sp. nov. and *L. dragescoi* sp. nov., the family Lacrymariidae is still monophyletic and the genus *Lacrymaria* is non-monophyletic, which is consistent with previous studies (Huang et al., 2018; Rajter et al., 2019; Wang et al., 2019).

*Lacrymaria songi* sp. nov. and *L. dragescoi* sp. nov. are both depicted in the core of *Lacrymaria* (Figure 7). *L. dragescoi* groups with *L. marina* population 1 and then clusters with *L. songi* sp. nov., which corresponds well with their morphological characteristics. With the addition of two new sequences, however, two populations of *L. marina* did not cluster together. Although the morphology of the two *L. marina* populations was not reported yet, we found that they are from different habitats, i.e., *L. marina* population 1 was collected from brackish water, while *L. marina* population 2 was collected from marine water. Therefore, both molecular data and the habitat imply that the two populations of *L. marina* are different species. Concerning the brackish habitat, *L. marina* population 1 is likely misidentified.

## Data availability statement

The datasets presented in this study can be found in online repositories. The names of the repository/repositories and accession number(s) can be found in the article/Supplementary material.

## Author contributions

JT: Investigation, Visualization, Writing – original draft, Writing – review & editing. GZ: Investigation, Validation, Writing – review & editing. JG: Writing – review & editing. LL: Writing – review & editing. JJ: Conceptualization, Supervision, Writing – review & editing. HP: Conceptualization, Funding acquisition, Supervision, Writing – review & editing.

## Footnotes

^1^http://guidance.tau.ac.il/ver2/

^2^http://www.phylo.org
