# Supplementary material for: A new contribution to the raptorial ciliate genus Lacrymaria (Protista: Ciliophora): a brief review and comprehensive descriptions of two new species from Changjiang Estuary
Source: Front Microbiol. 2023 Nov 6;14:1259653. doi: 10.3389/fmicb.2023.1259653 (PMC10658195; doi:10.3389/fmicb.2023.1259653)
Supplement: Supplementary file 1 [file Table_1.DOCX]

Supplementary Material

A new contribution to the raptorial ciliate genus *Lacrymaria* (Protista: Ciliophora): brief review and comprehensive descriptions of two new species from Changjiang Estuary

Jin Tang, Gongaote Zhang, Junqi Guo, Lingxuan Luo, Jiamei Jiang^*^, Hongbo Pan^*^

*** Correspondence:** Hongbo Pan, [hbpan@shou.edu.cn](mailto:hbpan@shou.edu.cn); Jiamei Jiang, [jm-jiang@shou.edu.cn](mailto:jm-jiang@shou.edu.cn)

# Supplementary Table and videos

**Table S1.** List of nominal species associated with the genus *Lacrymaria* and their current names and states.

| **Protonym** | **Current name** | **Species state** |
| --- | --- | --- |
| *Lacrymaria aciformis* Kahl, 1933 | *L. aciformis* | Maybe reliable |
| *Lacrymaria acuminata* Vuxanovici, 1962 | *L. acuminata* | Maybe reliable |
| *Lacrymaria acuta* Kahl, 1933 | *L. acuta* | Reliable |
| *Lacrymaria affinis* Bock,1952 | *L. affinis* | Maybe reliable |
| *Lacrymaria australis* Foissner & O'Donoghue, 1990 | *L. australis* | Reliable |
| *Lacrymaria balechi* Dragesco, 1960 | *Lacrymaria*? *balechi* | Maybe reliable |
| *Lacrymaria binucleata* Song & Wilbert, 1989 | *L. binucleata* | Reliable |
| *Lacrymaria buibosa* Alekperov, 1984 | *Lacrymaria*? *buibosa* | Unreliable |
| *Lacrymaria clavarioides* Alekperov, 1984 | *L. clavarioides* | Maybe reliable |
| *Lacrymaria caudata* Kahl, 1933 | *Phialina caudata* (Kahl, 1933) Wang, 2019 | Reliable |
| *Lacrymaria cohni* Buitkamp, 1974 | *L. cohni* | Reliable |
| *Lacrymaria conifera* Burkovsky, 1970 | *L. conifera* | Maybe reliable |
| *Lacrymaria coniformis* Penard, 1922 | *Lacrymaria*? *coniformis* | Maybe reliable |
| *Lacrymaria coronate* Claparède & Lachmann, 1858 | *P. coronate* (Claparède & Lachmann, 1858) Foissner, 1987 | Reliable |
| *Lacrymaria costata* Vuxanovici, 1963 | *L. costata* | Maybe reliable |
| *Lacrymaria cucumis* Penard, 1922 | *Lagunus cucumis* (Penard, 1922) Foissner, 1987 | Reliable |
| *Lacrymaria eylindriea* Vuxanovici, 1959 | *L. eylindriea* | Maybe reliable |
| *Lacrymaria decussata* Tucolesco, 1962 | *L. decussata* | Maybe reliable |
| *Lacrymaria delamarci* Dragesco, 1960 | *L. delamarci* | Maybe reliable |
| *Lacrymaria elegans* Engelmann, 1862 | *Lagynus elegans* (Engelmann, 1862) Quennerstedt, 1867 | Reliable |
| *Lacrymaria elongata* Vuxanovici, 1963 | *L. elongata* | Maybe reliable |
| *Lacrymaria exigua* Vuxanovici, 1962 | *L. exigua* | Reliable |
| *Lacrymaria filiformis* Foissner, 1983 | *L. filiformis* | Reliable |
| *Lacrymaria flagellifera* Gellert, 1957 | *L. flagellifera* | Maybe reliable |
| *Lacrymaria foliacea* Vuxanovici, 1962 | *L. foliacea* | Maybe reliable |
| *Lacrymaria fusus* Vuxanovici, 1962 | *L. fusus* | Maybe reliable |
| *Lacrymaria inflata* Vuxanovici, 1959 | *L. inflata* | Reliable |
| *Lacrymaria issykkulica* Alekperov, 1997 | *L. issykkulica* | Maybe reliable |
| *Lacrymaria kahli* Dragesco, 1960 | *L. kahli* | Reliable |
| *Lacrymaria lagenula* Clap. u. L., 1858 | *L. lagenula* | Reliable |
| *Lacrymaria lagynus* Gelei, 1954 | *L. lagynus* | Reliable |
| *Lacrymaria lata* Vuxanovici, 1962 | *L. lata* | Maybe reliable |
| *Lacrymaria lanceolata* Gelei, 1954 | *Phialina lanceolata* (Gelei, 1954) nov. comb. | Maybe reliable |
| *Lacrymaria longissima* Dragesco, 1966 | *L. longissima* | Maybe reliable |
| *Lacrymaria marina* Kahl, 1933 | *L. marina* | Reliable |
| *Lacrymaria maurea* Dragesco, 1965 | *L. maurea* | Maybe reliable |
| *Lacrymaria metabolica* Bünger, 1908 | *L. metabolica* | Maybe reliable |
| *Lacrymaria minima* Kahl, 1927 | *Phialina minima* (Kahl, 1927) Foissner, 2002 | Reliable |
| *Lacrymaria minuta* Dragesco, 1963 | *L. minuta* | Maybe reliable |
| *Lacrymaria monilata* Agamaliev，1966 | *L. monilata* | Maybe reliable |
| *Lacrymaria multinucleata* Dragesco, 1960 | *L. multinucleata* | Maybe reliable |
| *Lacrymaria nana* Song & Wilbert, 1989 | *L. nana* | Reliable |
| *Lacrymaria oblonga* Vuxanovici, 1962 | *L. oblonga* | Maybe reliable |
| *Lacrymaria olor* (Müller, 1786) Bory de Saint-Vincent 1824 | *L. olor* | Reliable |
| *Lacrymaria ovata* Burkovsky, 1970 | *Phialina ovata* (Burkovsky, 1970) nov. comb. | Maybe reliable |
| *Lacrymaria parva* Vuxanovici, 1962 | *L. parva* | Maybe reliable |
| *Lacrymaria perlucida* Vuxanovici, 1963 | *L. perlucida* | Maybe reliable |
| *Lacrymaria pulchra* Wenzel, 1953 | *L. pulchra* | Reliable |
| *Lacrymaria pumilio* Vuxanovici, 1962 | *L. pumilio* | Maybe reliable |
| *Lacrymaria pupula* Müller, 1786 | *Phialina pupula* (Müller, 1786) Song & Wilbert, 1989 | Reliable |
| *Lacrymaria rotundata* Dragesco, 1960 | *L. rotundata* | Maybe reliable |
| *Lacrymaria rostrata* Kahl, 1935 | *Pelagolacrymaria rostrata* (Kahl, 1935) Foissner, 1999 | Reliable |
| *Lacrymaria salinarum* Kahl, 1928 | *L. salinarum* | Reliable |
| *Lacrymaria sapropelica* Kahl, 1927 | *Lagynus sapropelica* | Maybe reliable |
| *Lacrymaria spiralis* Corliss, 1986 | *L. spiralis* | Maybe reliable |
| *Lacrymaria subsphaerica* Vuxanovici, 1962 | *L. subsphaerica* (Kahl, 1927) nov. comb. | Maybe reliable |
| *Lacrymaria trichocystus* Dragesco, 1960 | *L. trichocystus* | Maybe reliable |
| *Lacrymaria urnula* Kahl, 1930 | *Lagynus urnula* (Kahl, 1930) nov. comb. | Maybe reliable |
| *Lacrymaria vaginifera* Song & wilbert, 1989 | *L. vaginifera* | Reliable |
| *Lacrymaria vertens* Stokes, 1885 | *Phialina vertens* (Stokes, 1885) Foissner & Adam, 1979 | Reliable |
| *Lacrymaria vermicularis* Müller, 1786 | *Phialina vermicularis* (Müller, 1786) Bory de Saint-Vincent | Reliable |
| *Lacrymaria versatilis* Borror, 1963 | *L. versatilis* | Maybe reliable |
| *Lacrymaria vitrea* Vuxanovici, 1959 | *L. vitrea* | Reliable |

**Supplementary Video 1.** *Lacrymaria songi* sp. nov. is in the predatory state.

**Supplementary Video 2.** *Lacrymaria dragescoi* sp. nov. is in the predatory state.
